# Mechanism, spectrum, consequences and management of hyponatremia in tuberculous meningitis

**DOI:** 10.12688/wellcomeopenres.15502.2

**Published:** 2021-03-29

**Authors:** Usha K. Misra, Jayantee Kalita, Tuberculous Meningitis International Research Consortium

**Affiliations:** 1 Department of Neurology, Sanjay Gandhi Postgraduate Institute of Medical Sciences, Lucknow, India

**Keywords:** Tuberculous meningitis, hyponatremia, cerebral salt wasting, stroke, SIDH, natriuretic peptide

## Abstract

Hyponatremia is the commonest electrolyte abnormality in hospitalized patients and is associated with poor outcome. Hyponatremia is categorized on the basis of serum sodium into severe (< 120 mEq/L), moderate (120-129 mEq/L) and mild (130-134mEq/L) groups. Serum sodium has an important role in maintaining serum osmolality, which is maintained by the action of antidiuretic hormone (ADH) secreted from the posterior pituitary, and natriuretic peptides such as atrial natriuretic peptide and brain natriuretic peptide. These peptides act on kidney tubules via the renin angiotensin aldosterone system. Hyponatremia <120mEq/L or a rapid decline in serum sodium can result in neurological manifestations, ranging from confusion to coma and seizure. Cerebral salt wasting (CSW) and syndrome of inappropriate secretion of ADH (SIADH) are important causes of hyponatremia in tuberculosis meningitis (TBM). CSW is more common than SIADH. The differentiation between CSW and SIADH is important because treatment of one may be detrimental for the other; evidence of hypovolemia in CSW and euvolemia or hypervolemia in SIADH is used for differentiation. In addition, evidence of dehydration, polyuria, negative fluid balance as assessed by intake output chart, weight loss, laboratory evidence and sometimes central venous pressure are helpful in the diagnosis of these disorders. Volume contraction in CSW may be more protracted than hyponatremia and may contribute to border zone infarctions in TBM. Hyponatremia should be promptly and carefully treated by saline and oral salt, while 3% saline should be used in severe hyponatremia with coma and seizure. In refractory patients with hyponatremia, fludrocortisone helps in early normalization of serum sodium without affecting polyuria or functional outcome. In SIADH, V2 receptor antagonist conivaptan or tolvaptan may be used if the patient is not responding to fluid restriction. Fluid restriction in SIADH has not been found to be beneficial in TBM and should be avoided.

## Introduction

The human body is composed of 60–70% water, one-third of which is in the extracellular compartment. Sodium is the major electrolyte, which normally ranges between 135 and 145 mEq/L. Hyponatremia is defined as a serum sodium decrease of <135 mEq/L, and is the commonest electrolyte abnormality occurring in 3–35% of hospitalized patients, 50% of neurological admissions, and one-third of patients in intensive care units ^1^. Hyponatremia is a disorder of water balance. The severity of hyponatremia has been categorized as mild (130–134mEq/L), moderate (120–129 mEq/L) and severe (<120 mEq/L) ^2^, and serum sodium <125 mEq/L is regarded as an independent predictor of mortality, especially in critically ill patients;. In a review of hospitalized patients with hyponatremia, mortality increases by 1.5–60 times in the patients with hyponatremia compared to controls ^3^. Consequently, every attempt should be made to maintain a normal serum sodium level. It is important to check serum sodium levels twice to avoid laboratory error and use the lowest level to define the severity of hyponatremia. Hyponatremia in a patient may be due to a number of causes such as poor intake of sodium, drugs, vomiting, diarrhea, liver, kidney or heart failure, endocrine disorders, syndrome of inappropriate secretion of antidiuretic hormone (SIADH) and cerebral salt wasting (CSW). Serum osmolality is regulated by antidiuretic hormone (ADH) and kidney. Antidiuretic hormone is released from the posterior pituitary in response to an increase in serum osmolality. It is also released in response to reduced intravascular volume, although serum osmolality is the main trigger ^4^. ADH binds to ADH receptors in the kidney tubules, and results in re-absorption of water without re-absorbing sodium. An increase in ADH in the presence of normal or low serum osmolality is regarded as inappropriate, which results in continued absorption of water by the kidney resulting in hyponatremia and natriuresis. The kidneys are able to excrete sodium normally because sodium excretion is regulated by aldosterone and atrial natriuretic peptide (ANP). The main causes of hyponatremia are set out in Table 1. A number of neurological disorders such as stroke, subarachnoid hemorrhage, head injury, neurosurgical operations and central nervous system (CNS) infections may result in hyponatremia. This review will focus on the pathophysiology, diagnosis and management of hyponatremia with an emphasis in tuberculous meningitis (TBM).

## Pathophysiology of hyponatremia

Serum sodium has an important role in maintaining serum osmolality, and hyponatremia can be associated with normal, increased or reduced osmolality. In normal individuals, serum osmolality ranges between 280 mOsm/L and 295 mOsm/L, and is calculated by the following formula:

*Serum Osmolality = (Serum sodium x 2 + blood glucose/1.8 + blood urea/2.8) mEq/L*

The main causes of hyponatremia are set out in Table 1. There are two important causes of hyponatremia in neurological conditions: SIADH and CSW. 

**Table 1.** Causes of hyponatremia based on volume status.

Normovolemic	Hypovolemic	Hypervolemic
Endocrinal	Diabetes, corticosteroid withdrawal	Heart failure
Hypothyroidism	Sweating, burn	Chronic renal failure
Adrenal insufficiency	Ketone, urea	Cirrhosis of liver
Hypertonic fluid administration	Iatrogenic (hypotonic fluid)	Iatrogenic (hypertonic solution)
SIADH	CSW	SIADH

**SIADH** = syndrome of inappropriate antidiuretic hormone; **CSW** = cerebral salt wasting.

### Syndrome of inappropriate secretion of antidiuretic hormone (SIADH)

The underlying mechanism of SIADH is inappropriate release of ADH or arginine vasopressin resulting in low serum osmolality and water absorption. This leads to expansion of extra-cellular volume and dilutional hypotonic hyponatremia despite normal renal sodium handling. Although SIADH is a volume expanded state, most patients do not show the clinical evidence of hypervolemia, because only one-third of total retained water is in extracellular space. The causes of SIADH are as follows:

- *CNS disorders:* Meningitis, encephalitis, subarachnoid hemorrhage or trans-sphenoidal pituitary surgery.
- *Pulmonary disorders:* Pneumonia, bronchogenic carcinoma.
- *Malignancy*
- Surgery
- *Drugs:* carbamazepine, oxcarbazepine, cyclophosphamide, selective serotonin reuptake inhibitors

### Cerebral salt wasting (CSW)

CSW refers to primary natriuresis leading to hypovolemia and sodium depletion without known stimulus to excrete a large amount of sodium. It is suggested that natriuretic factors such as ANP, brain natriuretic peptide (BNP), C type natriuretic peptide and dendroaspis natriuretic peptide (DNP) may be responsible for CSW, although BNP is regarded as the most important cause of CSW ^4^. The release of ANP is mainly from cardiac atria and BNP from ventricles, hypothalamus, sympathetic projections and adrenal medulla. Release of ANP and BNP is mostly due to distension of the atria or ventricles in addition to various sympathetic and hormonal influences ^5, 6^. The effect of natriuretic peptides is well documented in nephrons, but less clear in the CNS and autonomic nervous system. It has, however, been suggested that dysregulation of the sympathetic response may be responsible for CSW; association of CSW with neuroleptic malignant syndrome suggests the role of the sympathoadrenal system and natriuretic peptides ^7^. A direct relationship between ANP and BNP with intracerebral pressure (ICP) has been reported ^4^. CSW may be a protective mechanism in response to excessive rise in ICP, vasospasm in subarachnoid hemorrhage or meningitis. Some studies, however, have not found such a direct relationship between BNP and CSW. In a study on TBM, ANP and BNP were elevated at the time of hyponatremia compared to basal values, and remained elevated even after correction of hyponatremia. ANP and BNP, however, did not differentiate between CSW and SIADH ^8^. The patients with SIADH had increased volume and sodium excretion in 24 hours compared to those without SIADH and subdural hemorrhage, but their BNP did not change and ANP decreased ^9^. In nine children with features of CSW, hyponatremia normalized by two weeks, but polyuria and natriuresis increased. The potential cause of CSW in these children was elevated ANP in 1 out of 6, and BNP in 2 out of 7 suggesting their limited role in CSW ^10^. Apart from ANP and BNP, other natriuretic peptides have also been studied. An elevated DNP level was associated with negative fluid balance and hyponatremia in patients with SIADH and head injury ^11, 12^. Dysregulated sympathetic activity may cause an increase in renal blood flow and glomerular filtration rate, and a decrease in renin release and renal tubular reabsorption ^13^.

## Clinical manifestations of hyponatremia

The clinical manifestations of hyponatremia are related to its severity and rate of decline in serum sodium. Symptoms generally appear when serum sodium decreases to 120 mEq/L or lower; however, a rapid decline in serum sodium may manifest at higher sodium level ^14, 15^. Headache, nausea, vomiting, anorexia, muscle cramps, myalgia, restlessness, confusion, lethargy and coma may ensue as serum sodium level declines. Neurological examination reveals changes in mentation and reduced tendon reflexes. In an advanced stage, cerebral edema develops, which may be associated with seizures, apnea, coma and death ^16^. In slowly developing hyponatremia, there may not be clinical symptoms and signs even with a very low serum sodium level, as the brain becomes adapted to hypo-tonicity by extruding solute to extracellular space. This process may ameliorate cellular swelling. The drawback of this adaptive process is that it may predispose to osmotic demyelination if hyponatremia is corrected rapidly. Osmotic demyelination typically affects pons and extra-pontine areas.

## Hyponatremia in tuberculous meningitis (TBM)

TBM is the commonest cause of sub-acute and chronic meningitis, and occurs in ~0.9% of the patients with tuberculosis. TBM is associated with basal exudates, hydrocephalous, tuberculoma and stroke, and is an important cause of stroke in young individuals in India ^17^. Hyponatremia in TBM is multifactorial and may be due to anorexia, nausea, vomiting, poor intake of sodium, diarrhea, drugs (diuretic, osmotic agents, carbamazepine, oxcarbazepine) and associated comorbidities.

Hyponatremia in TBM has been evaluated in only a few studies. In 20 children with TBM, hyponatremia was present in 65% on admission, 47% on day three and 30.8% on day 10. The cause of hyponatremia was diagnosed as SIADH. The outcome was not related to severity of meningeal inflammation. Two out of the 3 children who died within three days had SIADH ^18^. Another study in 115 TBM patients reported endocrinal dysfunctions in 53% and SIADH in 9.6% ^19^. In a prospective study on 76 TBM patients, 34 (44.7%) had hyponatremia, which was mild in 3, moderate in 23 and severe in 8 patients. CSW was the most frequent cause of hyponatremia in 17, SIADH in 3 and there were miscellaneous causes in 14 patients. Hyponatremia was related to the Glasgow Coma Scale score, severity of TBM, focal weakness, mechanical ventilation, age and comorbidities, while CSW was related to the severity of TBM ^20^. There are many short series and case reports on SIADH and CSW in TBM ^20– 22^. Studies that comprise of more than 10 patients have been included in Table 2. Out of a total of 11 studies comprising 642 (16–195 patients in each study) patients with TBM, 276 (44.3%) had hyponatremia. Only four studies, including 99 patients characterized CSW and SIADH, found CSW a more common cause of hyponatremia (36 patients; 36.4%) than SIADH (26 patients; 26.3%).

**Table 2.** Studies reporting hyponatremia in tuberculous meningitis patients.

Authors, year	Patients, n	Patients with hyponatremia, n (%)	Cause of hyponatremia, n (%)	Comments
^21^Lee *et al.,* 2018	TBM: 47; VM: 51	TBM: 37 (78.7); VM: 14 (27.5)		
^22^Inamdar *et al.,* 2016	75	29 (38.7)	CSW: 10; MISC: 19	No patients with SIADH
^20^Misra *et al., 2016*	76	34 (44.7)	CSW: 17; SIADH: 3; MISC: 14	No relationship reported to outcome
^23^Anderson *et al*., 2010	104	51 (49)		
^24^Smith *et al.,* 2000	20	12 (60)		
^18^Singh *et al.,* 1994	20	13 (65)	SIADH: 13	No effect on outcome after 72 hours
^25^Narotam *et al.,* 1994	24	15 (62.5)		Negative correlation between serum sodium with ANP and no correlation between plasma ADH and plasma sodium
^26^Shian *et al.,* 1993	16	11(70)		
^27^Davis *et al.,* 1993	54	43 (79)		
^28^Karandanis *et al.*, 1976	11	8 (73)		
^29^Bussmann *et al.,* 2001	195	20 (10.3)	SIADH: 7; CSW: 9	Hyponatremia attributable to CSW is at least as frequent in children as SIADH.
**Total**	**642**	**276 (43)**		
A summary of studies that had more than 20 patients and clearly defined CSW and SIADH criteria				
^22^Inamdar *et al.:* 2016	75	29		
^20^Misra *et al.:* 2016	78	34	CSW: 17; SIADH: 3	
^18^Singh *et al.:* 1994	20	6	CSW: 0; SIADH: 16	
^29^Bussmann *et al.:* 2001	195	20	CSW: 9; SIADH: 7	
**Total**	**366**	**99 (27.1)**	**CSW: 36 (36.4); SIADH:** **26 (26.3)**	

**CSW** = cerebral salt wasting; **MISC** = miscellaneous; **SIADH** = syndrome of inappropriate antidiuretic hormone; **ANP** = atrial natriuretic peptide; **ADH** = antidiuretic hormone, **VM**: viral meningitis,

### Relationship between hyponatremia and TBM-related stroke

**Figure 1.**
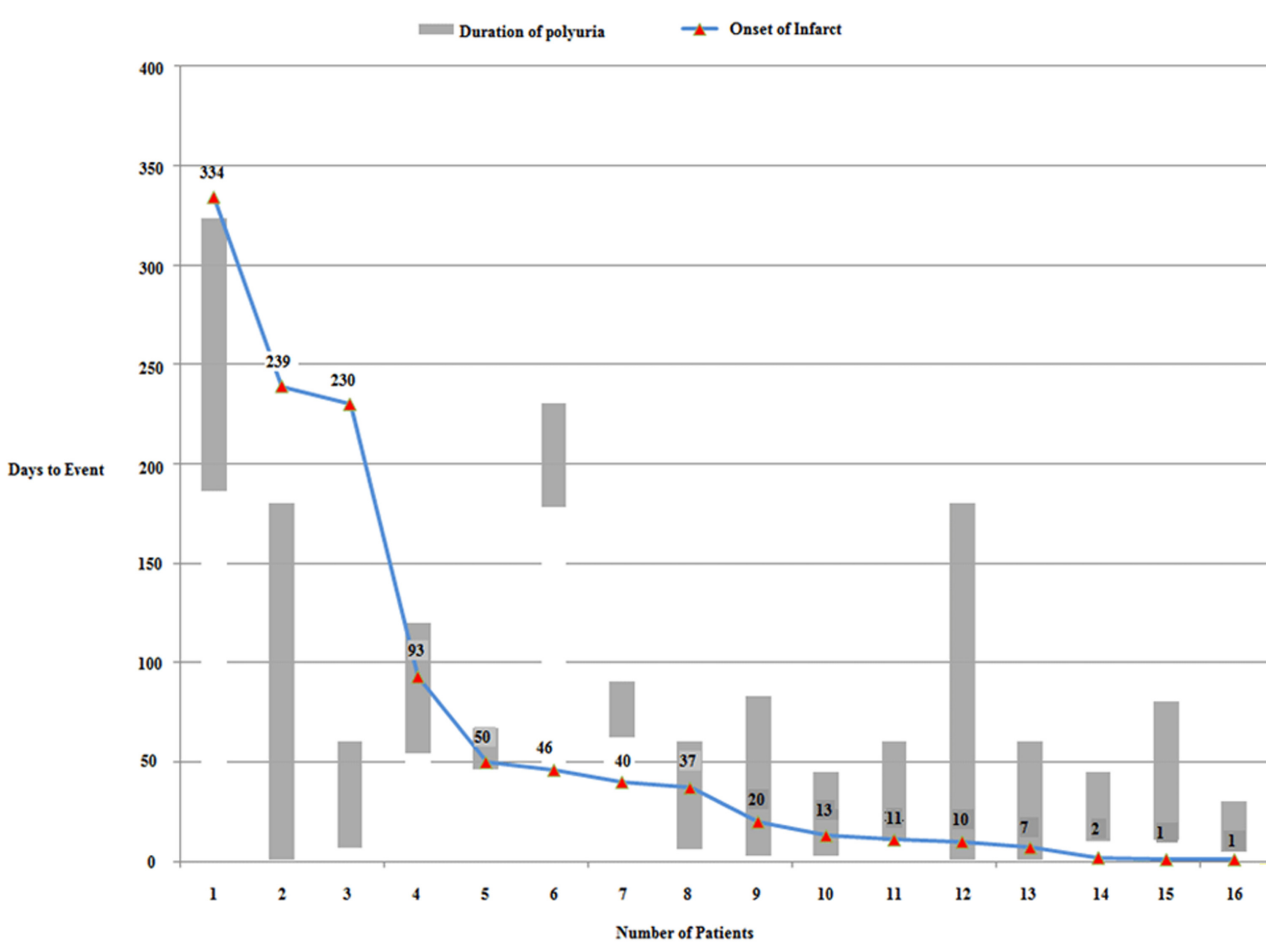
Duration of polyuria and onset of stroke in tuberculous meningitis patients with cerebral salt wasting (CSW). The vertical grey bars denote the onset (lower limit) and subsidence (upper limit) of polyuria in each patient. The red traingles denote the day of stroke after admission. A total of 10 out of 16 patients developed stroke during CSW (high urinary output). (Reproduced from Misra UK, Kalita J, Kumar M, *et al.*: Hypovolemia due to cerebral salt wasting may contribute to stroke in tuberculous meningitis. *QJM*. 2018; 111(7): 455–60. with permission).

**Figure 2.**
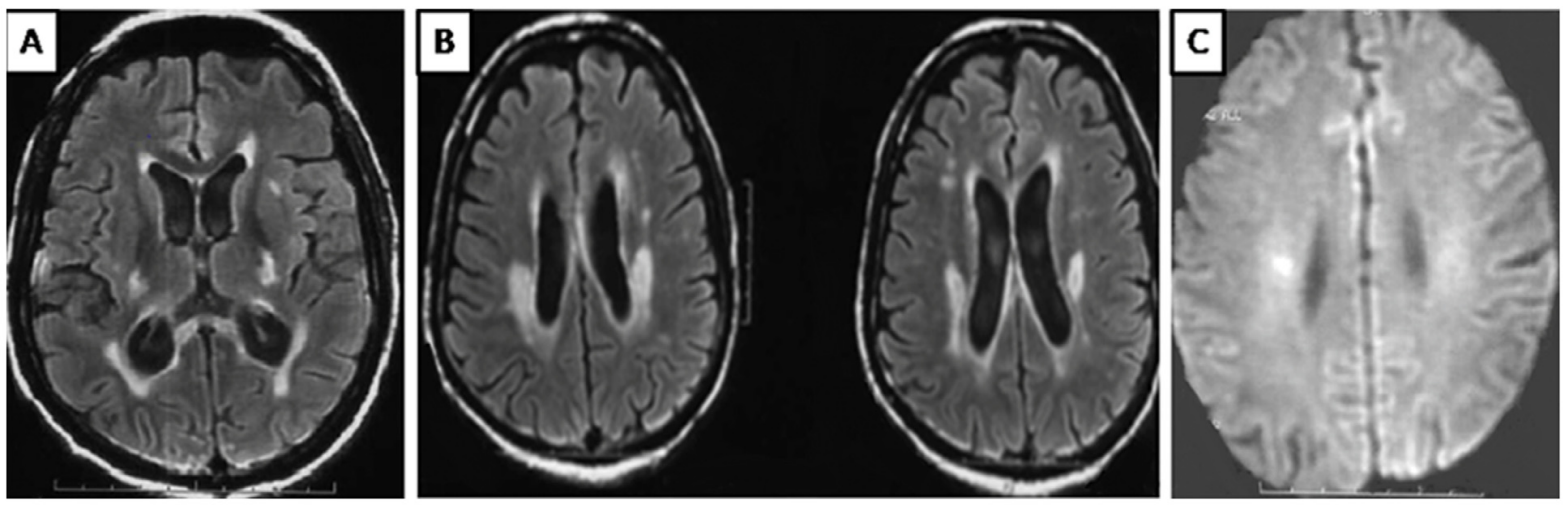
Cranial T2 FLAIR MRI axial sections in a 45- year-old male, stage III tuberculous meningitis (TBM) with type 2 diabetes mellitus and hypertension, and a 15-year-old male, stage III TBM patient. ( **A** and **B**) Infarcts are shown in the ( **A**) ischemic and ( **B**) peri ventricular region bilaterally (internal border zone) of the 45- year-old male. Cerebral salt wasting (CSW) was diagnosed on Day 40. The patient developed infarctions on Day 68 of admission. Hyponatremia was corrected after 12 days and urinary output normalized after 3 months. ( **C**) 15-year-old male showing asymptomatic infarct in right peri-ventricular white matter (internal border zone) with CSW diagnosed at admission (Day 1). (Reproduced from Misra UK, Kalita J, Kumar M, *et al.*: Hypovolemia due to cerebral salt wasting may contribute to stroke in tuberculous meningitis. *QJM.* 2018; 111(7): 455–60; with permission) ^38^.

Hyponatremia is reported in 40% of stroke patients ^30^ and up to 50% of TBM patients may have stroke ^31^. The relationship between TBM-related stroke and hyponatremia has been recently evaluated in a study of 81 patients with TBM, of which 32 (39.5%) had ischemic stroke. Stroke occurred at different time points: at the time of admission in 12 patients, within 3 months in 14 patients and after 3 months in 6 patients. Multiple infarctions were present in 20 (62.5%) patients, which were cortical in 7 and subcortical in 29 (capsular: 3, basal ganglia: 18, thalamus: 10, corona radiate: 13 and infra-tentorial: 4) patients. The infarctions were present in the tubercular zone in 10, ischemic zone in 15 and both in 7 patients. Hyponatremia occurred in 46 (57%) patients with TBM and was mainly due to CSW. A total of 16 patients with CSW had stroke, 10 of whom developed stroke during the poly-uric phase of CSW ( Figure 1). CSW patients with stroke had lower systolic blood pressure than those without CSW (115 vs 123 mm Hg; P = 0.04). Hyponatremia and polyuria were more severe and persisted for a longer time in stroke patients compared to those without stroke. Deep white matter infarction was more common in CSW ( Figure 2) compared to those without. It is possible that hypovolemia associated with CSW may result in hypo-perfusion and may contribute to infarction in a patient with basal exudate with compromised vascular lumen due to vasculitis. The additional contributing factors of stroke in TBM are endothelial injury due to vasculitis, prothrombotic state and strangulation of vessels by exudates ^31, 32^.

It is important to note that polyuria and negative fluid balance may persist for several months in TBM although hyponatremia improves earlier. Prolonged hypovolemia may lead to some beneficial (reducing intracranial pressure) and harmful effects (hypoperfusion and infarction). In TBM, the collaterals may also be affected, which are a natural defense mechanism to vascular occlusion, and internal border-zone may be more vulnerable in TBM ( Figure 1). In a previous study, internal border zone necrosis was reported in 50% children with TBM ^33^. There is a pressure gradient from the large artery to arterioles; blood pressure in brachial artery is 117/75mm Hg, thalamostriate artery 101/79 mm Hg, and perforators 59/38 mmHg ^34^. The pressure gradient in subcortical and perforators may render these regions especially vulnerable in the event of hypovolemia and hypotension associated with CSW. A dynamic state between lacunar infarction and white matter hyperintensity has been reported, leading to improvement or worsening in blood flow changes ^35^.

### Diagnosis of cause of hyponatremia in TBM

In a patient with hyponatremia, assessment of volume status is the most important step that differentiates SIADH from CSW ( Table 3). This differentiation is crucial because the treatment of one can be deleterious for the other condition. Clinical signs and laboratory results should be considered together to judge the volume status. Electrolytes and osmolality of serum and urine are important. Serum renin, ADH, ANP and BNP are not easily available, and usually do not differentiate CSW from SIADH. Serum potassium is normal in SIADH, but may be high in CSW. Serum uric acid is low in both SIADH and CSW, and on correction of serum sodium it rises in SIADH but remains low in CSW ^36, 37^. The definite diagnosis of the cause of hyponatremia may take some time, but empiric therapy may be started assuming CSW is more common and fluid restriction may be hazardous in CSW, especially in bacterial meningitis ^39– 42^.

**Table 3.** Differentiating features between CSW and SIADH.

Parameter	CSW	SIADH
Extracellular volume	↓	↑
Body weight	↓	↑
Fluid balance	Negative	Positive
Tachycardia	+	-
Hypotension	+	-
Hematoctrit/Blood urea nitrogen/Albumin	↑	Normal
Central venous pressure	↓	Normal or slightly high

**CSW** = cerebral salt wasting; **SIADH** = syndrome of inappropriate antidiuretic hormone.

CSW diagnosis should be considered in the presence of the following features:

*Essential:* (all required)

1. Polyuria (24 hour urine output > 3L for at least 2 consecutive days).
2. Hyponatremia: serum sodium < 135 mEq/L on 2 occasions 24 hours apart.
3. Exclusion of secondary causes of hyponatremia such as endocrine abnormalities, renal, cardiac or hepatic failure, or diuretics.

*Supportive criteria* *(at least 3 out of 5):*

1. Clinical evidence of hypovolemia such as hypotension, dry mucous membrane, tachycardia or postural hypotension.
2. Persistently negative fluid balance as revealed by intake output chart and/or weight loss.
3. Laboratory evidence of dehydration such as elevated hematocrit, hemoglobin, serum albumin or blood urea nitrogen.
4. Central venous pressure (CVP) < 6 cm of water.
5. Urinary sodium > 40 mEq/L or urine osmolality > 300 mOsm/L on 2 consecutive occasions

Diagnosis of SIADH is based on the following criteria ^43^:

1. Hyponatremia
2. Low serum osmolality
3. High urinary osmolality > 100mOsm/Kg.
4. Urinary sodium > 20mMol/L
5. Exclusion of endocrinal diseases, renal causes, and disorders of non-osmotic release of ADH such as hypovolemia, hypotension, pain, stress, drugs (narcotic, carbamazepine, cyclophosphamide, selective serotonin reuptake inhibitors)

Daily sodium balance, intake-output and body weight chart should be maintained. When hyponatremia is refractory to IV saline and oral salt; water and salt intake should be carefully increased after reassessing the diagnosis.

Some tests have been recommended to differentiate CSW from SIADH:

- *Frusemide test:* Infusion of 20mg of frusemide normalizes serum sodium in the patients with SIADH ^44^
- *Saline infusion test:* Hyponatremia is aggravated after infusion of 100 ml of normal saline in SIADH

SIADH and CSW may have overlapping clinical and laboratory features such as hyponatremia, low serum osmolality, high urinary sodium and osmolality. The most reliable differentiating feature is evidence of low extra cellular volume in CSW, which is normal or increased in SIADH.

In SIDAH, hyponatremia is aggravated by infusing 100 mL of normal saline, whereas it leads to normalization of serum sodium in CSW. Similarly, 20 mg of furosemide may normalize serum sodium in SIADH but not in CSW ^5^. However, the safety or reproducibility of these tests has not been validated. Hence, we have not used saline infusion test ^44^. Serum uric acid is likely to be low in SIADH and normal or high in CSW. This relationship has, however, not been well studied. In a study, serum uric acid was unexpectedly low in CSW ^3^. Hyponatremia and high urate excretion might be a common feature of intracranial disease in general ^45^.

Some authors do not differentiate between CSW and SIADH and have suggested a term ‘hyponatremia natriuretic syndrome’ ^25^ or ‘cerebral wanting syndrome’ ^46^. However, using the simple bedside criteria stated above, the authors of the present article feel comfortable in differentiating CSW from SIADH.

## Management of hyponatremia in TBM

### Asymptomatic hyponatremia

In a patient with asymptomatic hyponatremia with volume contraction, ADH level is increased as a compensatory response. Normal saline should be administered to restore intravascular volume and free water should be avoided. As the intravascular volume is normalized, the stimulus for ADH release is eliminated and excess water is excreted leading to correction of hyponatremia. In CSW, polyuria continues and fluid has to be administered as long as hyponatremia persists. In patients with SIADH, fluid restriction may be sufficient.

### Symptomatic hyponatremia

**Figure 3.**
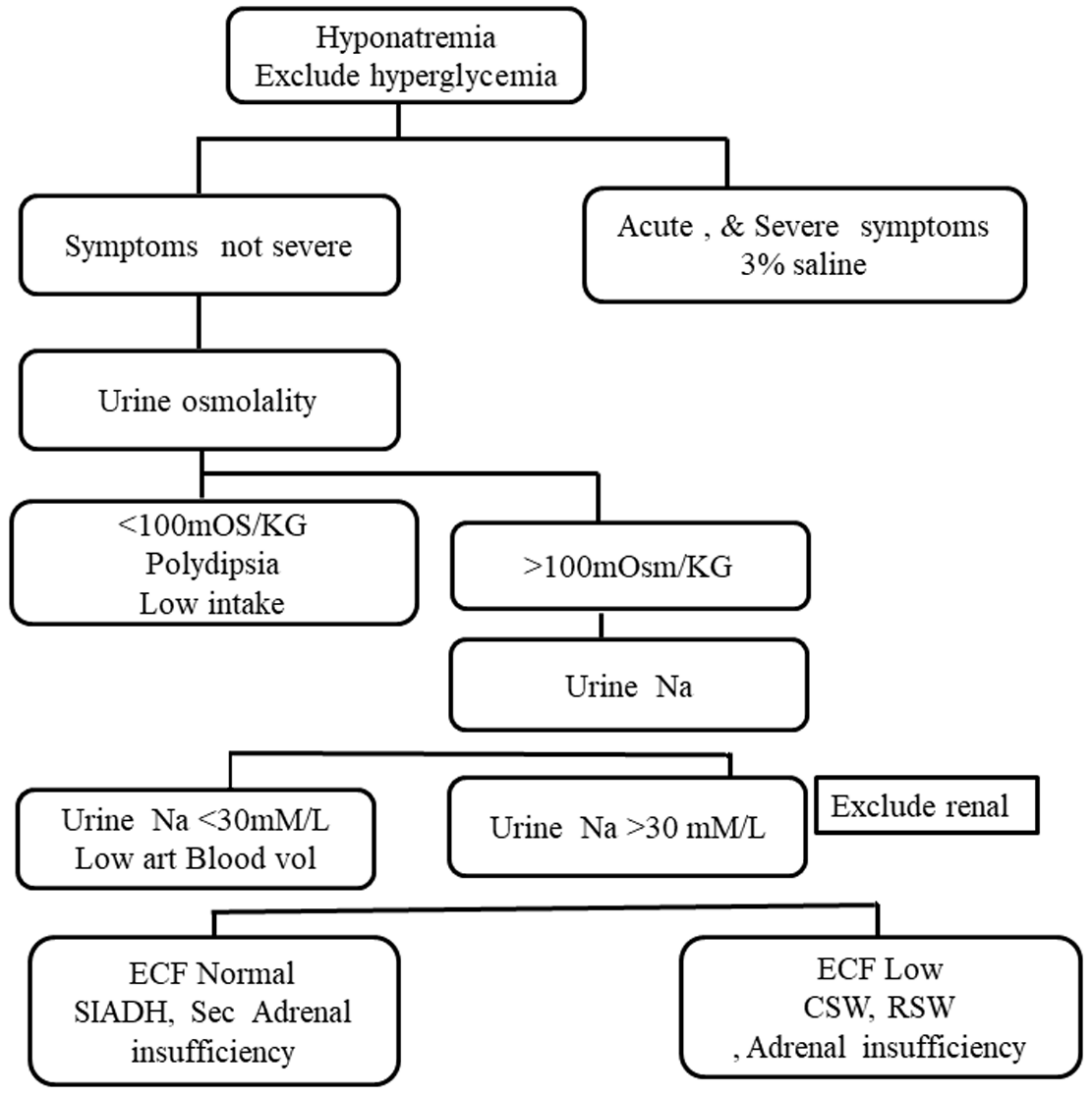
Schematic diagram shows management of hyponatremia. art= arterial.

Management of hyponatremia: In TBM, there is limited information about the management hyponatremia. There is need to generate more information to rationalize the management of hyponatremia in TBM. However, the management of hyponatremia in TBM is based on the available evidences in other conditions. Urgent treatment of hyponatremia may be lifesaving, although sometimes a proper assessment of the patient may not be possible because of shock, sedation or mechanical ventilation in severe meningitis. Mild hyponatremia may corrected by addition of 10–12gm salt/day. In severe or moderately severe hyponatremia, the risk of brain edema may outweigh that of osmotic demyelination. Urgent treatment of hyponatremia is needed in patients with severe clinical manifestations such as coma, convulsions, vomiting, cardiorespiratory distress, or deep somnolence. Treatment is initiated with 3% saline at a rate of 0.5–2ml/kg/hour till symptoms resolve; but resolution of symptoms may nor occur in TBM. The rate of sodium correction should not exceed by 10 mEq/L in first 24 hours, and 8 mEq/L in the next 24 hours (Braun *et al.*, 2015; Sterns *et al.*, 2009 ^46, 47^. As per European Guideline, 150ml of 3% saline is administered in 20 min and serum sodium is checked every 20min. 3% saline should be repeated till target rise of serum sodium of 5mEq/L is achieved ^48^. Frequent monitoring of serum sodium is required to avoid rapid correction and central pontine myelinolysis (CPM). If there is no symptomatic improvement after raising serum sodium by 5 mEq/L during first hour, other causes should be checked including imaging ^48^. The management of hyponatremia is presented in Figure 3.

In resource poor setting or if the facilities for frequent serum sodium are not available, one may start with 3% saline, following clinical improvement normal saline and oral salt (10–12 g/day) as salt capsule or through nasogastric tube may be administered. Serum sodium may be monitored as frequently as possible based on above mentioned guidelines. 

### Fludrocortisone (FC)

There is inhibition of renin angiotensin-aldosterone system in CSW; therefore, FC has been used in patients who are refractory to saline and oral salt treatment. There are however very few studies evaluating the role of FC in CSW. In a randomized controlled trial in SIADH, FC resulted in restoration of sodium balance and reduction in delayed stroke ^49^. In TBM, the role of FC in CSW was initially based on an isolated case report or short series ^50– 53^. In a recent randomized controlled trial of patients with TBM-associated CSW, 18 patients each were randomized to oral FC (0.4-1 mg daily) and no FC groups. In addition, both the groups received normal saline and oral salt (5–12g/d). Serum sodium level was normalized earlier in the FC group compared to the no-FC group (4 vs 15 d; P = 0.04). Hospital morality and 3 and 6 month disability did not differ, but there were fewer infarctions in internal border zone in the FC group (6% vs 33%; P = 0.04). FC was associated with severe hypokalemia and hypertension in two patients each and pulmonary edema in one patient. In two patients, FC had to be withdrawn because of adverse events. This study concluded that FC results in earlier normalization of serum sodium and fewer infarctions in deep white matter in patients with TBM-related CSW. Polyuria however was not influenced by FC ^54^.

### V2 receptor antagonists

Arginine vasopressin peptide receptor antagonist- intravenous conivaptan and oral tolvaptan have been used in the management of hyponatremia in SIADH. The V2 receptor antagonists bind to V2 receptors in the collecting tubule of the kidney and prevent binding of ADH. This results in excretion of water (aquaresis) leading to increased urinary output and decreased urinary tonicity. Both conivaptan and tolvaptan have been studied in patients with SIADH ^55– 57^ and are both effective in increasing serum sodium. The dose of tolvaptan is 15, 30, or 60 mg depending on serum sodium level. Side effects of tolvaptan include dryness of mouth, increased thirst, constipation and polyuria ^55^. Conivaptan is administered as 20mg IV over 30 min followed by continuous infusion of 20–40mg up to 96 hours. Adverse reactions of conivaptan are local reaction, edema, hypokalemia, increased urinary output and increased thirst ^55^. A review of 20 trials including 2900 patients comparing vasopressin receptor antagonist versus placebo in mild to moderate hyponatremia did not reduce mortality, RR 1.0 8(95% CI 0.08-1.46). A subgroup analysis revealed that there was insignificantly increased risk of death in the patients with hypervolemia ^58^. Updated analysis of trials after that by Resen Zvi revealed that vasopressin receptor antagonists resulted in increased serum sodium at 3–7 days and 7 months. There was no difference in adverse events but the risk of rapid rise in serum sodium was 60% higher in VRA group compared to placebo (RR 1.61, 85% CI 1.11 - 2.33) and were consistent in various VRAs (tolvaptan, conivaptan, lixivaptan, and satavaptan) though no patient developed CPM. Subsequently, there are reports of CPM leading to neurological sequelae in patients receiving tolvaptan ^59^, leading to drug safety communication by FDA ^60^. There are also concerns about high alanine aminotransferase (4.4% vs 1%) ^61^. Vasopressin antagonists are contraindicated in CSW.

## Conclusion

Hyponatremia is common in TBM and occurs most frequently due to CSW. Volume contraction associated with CSW may contribute to border zone infarction. Fludrocortisone treatment may normalize serum sodium earlier than those on saline and salt treatment only, but polyuria persists. Further studies are needed to develop strategies to manage volume contraction in CSW. 

## Data availability

No data are associated with this article.
