# Supplementary material for: Mechanism, spectrum, consequences and management of hyponatremia in tuberculous meningitis
Source: Wellcome Open Res. 2021 Mar 29;4:189. Originally published 2019 Nov 28. [Version 2] doi: 10.12688/wellcomeopenres.15502.2 (PMC7372311; doi:10.12688/wellcomeopenres.15502.2)
Supplement: Peer review history

# Peer review: Reviewer response for version 1

**DOI:** 10.21956/wellcomeopenres.16964.r38779

**Published:** 2020-07-20

**Authors:** Ghulamullah Lail

**Affiliations:** 1 Department of Medicine and Allied, Jinnah Medical and Dental College, Karachi, Pakistan

Dr Misra and Dr Kalita excellently reviewed a relatively common topic: “Hyponatremia in tuberculous meningitis (TBM)”. This review is clear and concise takes in consideration broader viewers almost everyone graduate, postgraduates fellows and seniors may be benefited. Hyponatremia is commonly encountered in our daily clinical practice. Authors have summarized here the basics including pathogenesis, clinical features diagnosis and treatment. Possible reasons of hyponatremia in TBM discussed here with much focus on cerebral salt wasting (CSW) and syndrome of inappropriate antidiuretic hormone secretion (SIADH). Early recognition and prompt response improve outcomes in such situation.

 Authors are knowledgeable and given precise review. I have gone through previous peer review report, valuable comments given from Parveen Kumar Sharma, previous peer review has not changed my decision making. Here are few humble suggestion from my side.

**Abstract**:

 “In SIADH, V2 receptor antagonist conivaptan or tolvaptan may be used if the patient is not responding to fluid restriction. Fluid restriction in SIADH has not been found to be beneficial in TBM and should be avoided.” These sentences gives reader a thought that in TBM patients SIADH must be managed by vaptans. (First sentence emphasizes if fluid restriction doesn't benefit vaptans may be given, in next sentence recommends avoid fluid restriction in TBM) so a brief overview of the vaptans better be included in abstract. (Spasovski, Eur J Endocrinology 2014 ^1^).

 Table 2: Shows two totals 642 and 366 reason for two totals is not explained in foot notes of table.

 Diagnostic criteria for SIADH may be adjusted as more than 30mmol/l (J AmSocNephrol 28: 1340–1349, 2017 ^2^) higher urate clearance observed during hyponatremia related to SIADH(CJASN July 2008, 3 (4) 1175-1184 ^3^)

 No reference given for saline infusion test.

 “the authors of the present article feel comfortable in differentiation CSW and SIADH.” Better if this sentence is supported by scientific evidence like sensitivity and specificity considering authors used these simple bedside tests.

**Management of hyponatremia in TBM**

 In asymptomatic patients with SIADH Fluid restriction may be sufficient. While in abstract it is

 “Fluid restriction in SIADH has not been found to be beneficial in TBM and should be avoided”.

 Symptomatic patients; Hypertonic saline infusion rate not explained.  Hypertonic saline (2mL/kg) indicated. (Spasovski, Eur J Endocrinology 2014 ^1^).

 I hope these small comments will be helpful for the review paper.

Is the review written in accessible language?

Yes

Are all factual statements correct and adequately supported by citations?

Yes

Are the conclusions drawn appropriate in the context of the current research literature?

Yes

Is the topic of the review discussed comprehensively in the context of the current literature?

Yes

Reviewer Expertise:

NA

I confirm that I have read this submission and believe that I have an appropriate level of expertise to confirm that it is of an acceptable scientific standard.

## References

[1] : Clinical practice guideline on diagnosis and treatment of hyponatraemia. *Eur J Endocrinol*.2014;170(3) : 10.1530/EJE-13-1020 G1-47 10.1530/EJE-13-1020 24569125

[2] : Diagnosis and Treatment of Hyponatremia: Compilation of the Guidelines. *J Am Soc Nephrol*.2017;28(5) : 10.1681/ASN.2016101139 1340-1349 10.1681/ASN.2016101139 28174217PMC5407738

[3] : Clinical laboratory evaluation of the syndrome of inappropriate secretion of antidiuretic hormone. *Clin J Am Soc Nephrol*.2008;3(4) : 10.2215/CJN.04431007 1175-84 10.2215/CJN.04431007 18434618

# Response

**Published:** 2021-02-11

**Authors:** USHA KANT MISRA

**Affiliations:** SGPGIMS, India

Dear Sir,

 The above-mentioned changes have been included in the revised version and resubmitted for publication in your esteemed journal.

**Ghulamullah Lail**

1. Fluid restriction and Vaptans in SIADH in TBM.

**Answer:** In our experience CSW is much more common than SIADH. In view of hazards of fluid restriction in meningitis, we feel that the patients should be treated with hypertonic saline during emergency which may be followed by normal saline and salt. If one is very sure of the diagnosis, vaptans may be used but in our experience it is not common. We have included discussion on vaptans in the paper. 

1. Diagnostic criteria for SIADH may be adjusted as more than 30mmol/l (J AmSocNephrol 28: 1340–1349, 2017 ) higher urate clearance observed during hyponatremia related to SIADH (CJASN July2008, 3 (4) 1175-1184)

**Answer:** We have kept the diagnosis of CSW and SIADH based on simple bedside criteria avoiding lab estimations which may not be widely available. We agree that the utility of our criteria need to be confirmed  by other investigators.

1. No reference given for saline infusion test.

**Answer:** In SIDAH, hyponatremia is aggravated by infusing 100 mL of normal saline, whereas it leads to normalization of serum sodium in CSW. Similarly, 20 mg of furosemide may normalize serum sodium in SIADH but not in CSW.5 However, the safety or reproducibility of these tests has not been validated. Hence, we have not used saline infusion test (Casulari et al 2004)

# Peer review: Reviewer response for version 1

**DOI:** 10.21956/wellcomeopenres.16964.r37864

**Published:** 2020-02-24

**Authors:** Praveen Kumar Sharma

**Affiliations:** 1 Department of Neurology, King George Medical University (KGMU), Lucknow, Uttar Pradesh, India

This article gives concise and very informative coverage of all aspects of hyponatremia, a commonly encountered problem in patients of tuberculous meningitis. Dr. Misra et al. provided some basics pertaining to hyponatremia followed by its pathophysiology in terms of any underlying cause and clinical features along with the importance of its early recognition and timely treatment to avoid the poor outcome. Subsequently, authors have summarized knowledge about hyponatremia associated with TBM which included a brief review of previous studies, its relationship with stroke, an approach to diagnosing the cause of hyponatremia and treatment. This article also tells about the importance of differentiating CSW from SIADH to avoid incorrect and at times potentially deleterious treatment.

 Overall, this article is clearly written, concise, crisp and gives valuable knowledge for practitioners to apply in the management of the patients of TBM to improve outcomes as well as for researchers to find new avenues. I have the following small comments to consider.

1. Hypovolemia is a cardinal feature of CSW, and it acts as a stimulus for ADH secretion too. So, there is a theoretical possibility as well as studies also to support that sometimes they both coexist in the same patient wherein the cause is labelled as mixed. Possibly, miscellaneous causes of hyponatremia in a few previous studies as summarized here might have included mixed cases too ^1^^,^^2^. I suggest little elaboration of the mixed cause and strategy of its management should have been included as well.
2. Exclusion of endocrine dysfunction is important for the diagnosis of both CSW and SIADH. Here I wish to add since endocrine dysfunction is not uncommon in TBM and can be central in origin, Hypercortisolism and Hypothyroidism which can cause hypo-osmolar hyponatremia, should have been stressed upon for the exclusion with appropriate tests ^3^.
3. I suggest including fractional excretion of Urate as lab support for the diagnosis of SIADH since the criteria given here for its diagnosis are fine in a clearly hypervolemic patient but become confusing in euvolemic mildly hypervolemic cases ^4^.

At the last, I must say that Dr. Misra et al. have given a very clear view of a complicated but frequently encountered problem in clinical practice particularly in tropical countries like India. This is going to be of immense value to the practitioners and food for thought to researchers.

Is the review written in accessible language?

Yes

Are all factual statements correct and adequately supported by citations?

Yes

Are the conclusions drawn appropriate in the context of the current research literature?

Yes

Is the topic of the review discussed comprehensively in the context of the current literature?

Yes

Reviewer Expertise:

Epilepsy, Stroke & Neuro-infections

I confirm that I have read this submission and believe that I have an appropriate level of expertise to confirm that it is of an acceptable scientific standard.

## References

[1] : The incidence and pathophysiology of hyponatraemia after subarachnoid haemorrhage. *Clin Endocrinol (Oxf)*.2006;64(3) : 10.1111/j.1365-2265.2006.02432.x 250-4 10.1111/j.1365-2265.2006.02432.x 16487432

[2] : Concurrence of Inappropriate Antidiuretic Hormone Secretion and Cerebral Salt Wasting Syndromes after Traumatic Brain Injury. *Frontiers in Neuroscience*.2017;11: 10.3389/fnins.2017.00499 10.3389/fnins.2017.00499 28932182PMC5592206

[3] : Endocrine disorders: causes of hyponatremia not to neglect. *Ann Med*.2011;43(3) : 10.3109/07853890.2010.530680 179-87 10.3109/07853890.2010.530680 20964584

[4] : Syndrome of Inappropriate Antidiuresis. *Pediatr Clin North Am*.66(1) : 10.1016/j.pcl.2018.09.005 209-226 10.1016/j.pcl.2018.09.005 30454744

# Response

**Published:** 2021-02-11

**Authors:** USHA KANT MISRA

**Affiliations:** SGPGIMS, India

Dear Sir,

 The above-mentioned changes have been included in the revised version and resubmitted for publication in your esteemed journal.

  

1. elaboration of the mixed cause and strategy of its management should have been included as well.

**Answer:** The miscellaneous causes refer to other causes of hyponatremia and our comments are targeted to clearly defined cases. Using our criteria we feel that a clear diagnosis is possible. However some authors have emphasized that it is difficult to differentiate these conditions clearly and they suggest the term salt wanting syndrome   and treat it with saline when in doubt.

1. Exclusion of endocrine dysfunction is important for the diagnosis of both CSW and SIADH. Here I wish to add since endocrine dysfunction is not uncommon in TBM and can be central in origin, Hypercortisolism and Hypothyroidism which can cause hypo-osmolar hyponatremia, should have been stressed upon for the exclusion with appropriate tests .

**Answer:** We agree that endocrinal disorders should be excluded before diagnosis of CSW and SIADH. If present these have to be managed as usual.

1. Suggest including fractional excretion of Urate as lab support for the diagnosis of SIADH since the criteria given here for its diagnosis are fine in a clearly hypervolemic patient but become confusing in euvolemic mildly hypervolemic cases

**Answer:** Serum uric acid is likely to be low in SIADH and normal or high in CSW. This relationship has, however, not been well studied. In a study, serum uric acid was unexpectedly low in CSW.3 Hyponatremia and high urate excretion might be a common feature of intracranial disease in general (Maesaka et al 1998 )
